# Gene and whole genome analyses reveal that the mycobacterial strain JS623 is not a member of the species *M* *ycobacterium smegmatis*

**DOI:** 10.1111/1751-7915.12336

**Published:** 2016-02-01

**Authors:** Maria J. Garcia, Susanne Gola

**Affiliations:** ^1^ Universidad Autonoma de MadridMadridSpain; ^2^ Centro Nacional de Biotecnología (CNB‐CSIC)MadridSpain

## Abstract

Unexpected differences were found between the genome of strain JS623, used in bioremediation studies, and the genome of strain mc^2^155, a model organism for investigating basic biology of mycobacteria. Both strains are currently assigned in the databases to the species *M* *ycobacterium smegmatis* and, consequently, the environmental isolate JS623 is increasingly included as a representative of that species in comparative genome‐based approaches aiming at identifying distinctive traits of the different members of the genus *M* *ycobacterium*. We applied traditional molecular taxonomic procedures – inference of single and concatenated gene trees – to re‐evaluate the membership of both strains to the same species, adopting the latest accepted cut‐off values for species delimitation. Additionally, modern whole genome‐based *in silico* methods where performed in a comprehensive molecular phylogenetic analysis of JS623 and other members of the genus *M* *ycobacterium*. These analyses showed that all relevant genome parameters of JS623 clearly separate this strain from *M* *. smegmatis*. The strain JS623 should be corrected as *M* *ycobacterium* sp. not only in the literature but, even more importantly, in the database entries, as inclusion of the genome wrongly attributed to the *M* *. smegmatis* species in comparative studies will result in misleading conclusions.

## Introduction

*Mycobacterium smegmatis* (Trevisan) Lehmann and Neumann 1899 is a typical environmental rapid‐growing mycobacterium (Kazda *et al*., 2009) that occasionally causes infections in animals and humans (Vonmoos *et al*., 1986; Bercovier and Vincent, 2001; Brown‐Elliott and Wallace, 2002). It gained importance in the tuberculosis research field as a non‐pathogenic model system for certain aspects of mycobacterial biology (Reyrat and Kahn, 2001; Shiloh and Champion, 2010) and as a working‐horse for the development of new molecular genetic tools (e.g. Ehrt *et al*., 2005; Boldrin *et al*., 2010). The subsequent application of these tools in *Mycobacterium tuberculosis* greatly enhanced our knowledge on the pathogen in the recent past. Besides, *M. smegmatis* is also an interesting model on its own right. Its investigation stimulates (myco) bacterial research with basic discoveries, as the identification of a novel conjugation mechanism (Mizuguchi and Tokunaga, 1971; Gray *et al*., 2013), and impacts on state‐of‐the‐art technologies, as illustrated by the utilization of the *M. smegmatis* porin A in nanopore sequencing procedures (Derrington *et al*., 2010). The use of *M. smegmatis* as referential non‐pathogenic mycobacterium has been boosted by the arrival of whole genome sequence (WGS) technologies.

The type strain, *M. smegmatis* ATCC 19420, is an offspring of the ancient isolate of Alvarez and Tavel, who recovered the microorganism from smegma (http://www.bacterio.net/mycobacterium.html). In spite of its role as type strain (which means that it is the reference to which all candidate strains have to be compared with in order to determine whether they belong to the *M. smegmatis* species), the genome of ATCC 19420 has not been completed yet. On the contrary, the genome of several other *M. smegmatis* strains, such as mc^2^155 and JS623, has been sequenced recently. The former is the most widely used strain for genetic approaches within the genus because it is easily transformable with replicating plasmids, the latter is an environmental isolate from soil, used in bioremediation (Coleman and Spain, 2003; Jin and Mattes, 2008; Jin *et al*., 2010). Strain JS623 became popular after publication of its complete genome sequence. It can be foreseen that these strains will be increasingly included in comparative genome‐based studies in the mycobacterial field, both under genetic and environmental aspects.

In the context of a general, taxonomically motivated, approach to the genus, a preliminary study on the use of WGS data for the classification of mycobacteria was undertaken. In those studies, we stumbled upon discrepancies when comparing strain JS623 with the strain mc^2^155. That finding was unexpected, because both strains appear labelled as *M. smegmatis* in main molecular databases (Table 1).

**Table 1:** Databases that include JS623 labelled as *M* *. smegmatis*

Database*	Web‐site link to JS623 information
JGI	http://genome.jgi‐psf.org/mycsm/mycsm.info.html
KEGG	http://www.genome.jp/kegg‐bin/show_organism?org=msa
NCBI	http://www.ncbi.nlm.nih.gov/genome/genomes/1026
PATRIC	https://www.patricbrc.org/portal/portal/patric/Genome?cType=genome&cId=710686.3
UniProt	http://www.uniprot.org/taxonomy/710686

* Joint Genome Institute (JGI), Kyoto Encyclopedia of Genes and Genomes (KEGG), National Center for Biotechnology Information (NCBI), Pathosystems Resource Integration Center (PATRIC), Universal Protein Resource (UniProt).

At least two recent other studies, which followed the rationale to cover a broad range of mycobacterial genomes for comprehensive comparative analyses, included the strain JS623 and detected differences, compared with *M. smegmatis*, for single traits: the screening of mycobacterial genomes for prophages, identified an prophage‐like element specific in JS623 (Fan *et al*., 2014), and Osório and colleagues, interested in the distribution of L‐lactate dehydrogenase genes among mycobacteria, found different numbers of *lld*D paralogues in JS623 compared with other *M. smegmatis* strains (N.S. Osório, pers. comm.).

To find out whether the origins of the *M. smegmatis* strains with available WGS data could offer an explanation for the detected differences, the history of the different isolates was traced back (see Supporting Information). Yet, historical data did not allow a clear allocation of the strain JS623 as a member of the species *M. smegmatis*.

To clarify if the strain JS623 was or was not a member of the species *M. smegmatis*, phylogenetic relationships were inferred from genotypes by traditional approaches (16S rRNA gene and concatenated housekeeping genes similarities) and three WGS based methods.

## Results and discussion

### Gene‐scale comparisons

The availability of genome sequences allows now also for mycobacteria to infer phylogenetic relationships based on the 16S rRNA gene that are not only sustained by the similarity of the hypervariable regions but the whole coding sequence, leading to more precise results (Tortoli, 2012; Yarza *et al*., 2014).

In order to cover a broad range of fast‐growing *Mycobacterium* species, analysing at the same time the longest 16S rRNA coding region possible, the sequences from 22 representative rapid growers with approved names (http://www.bacterio.net/mycobacterium.html) were obtained from the NCBI genome database during October 2014. In cases when the genome has not been annotated yet, a 16S rRNA gene sequence was sought manually in the scaffolds or contigs (Supporting Information Table S1). Additionally, the 16S rRNA gene sequences from *M. goodii*, *M. moriokaense* and *M. wolinskyi* were obtained from GenBank (Supporting Information Table S2).

**Figure 1:**
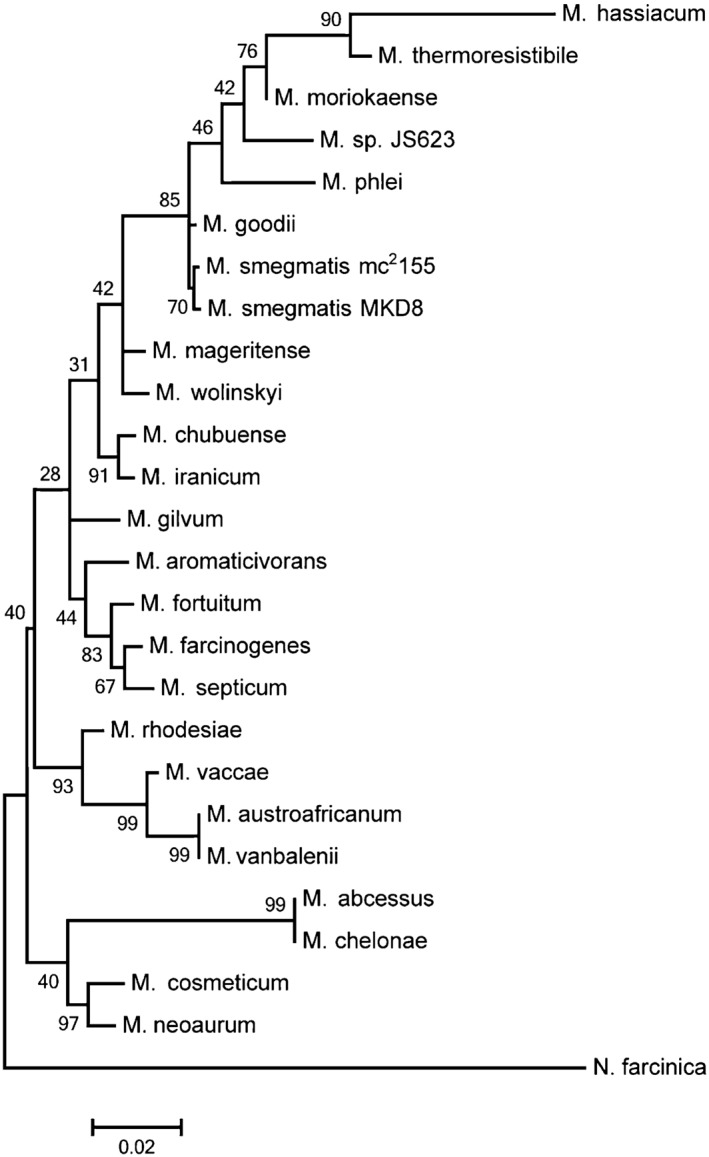
Molecular phylogenetic analysis based on the 16S rRNA coding sequence. Numbers are the percentage of trees in which the associated taxa clustered together in the bootstrap analysis. The tree is drawn to scale and the bar length indicates 0.02 substitutions per site. See Supporting Information Table S1 for accession number of the 16S rRNA genes and text and Experimental Procedures in Supporting Information for details.

Figure 1 shows the relationship of strain JS623 to other rapid‐growing mycobacteria, based on the analysis of the nearly full‐length 16S rRNA coding sequence. Strain JS623 clusters within the group of the so‐called thermo‐tolerant rapid growers (Tortoli, 2003), but is clearly distant from the two other *M. smegmatis* strains included in the tree, as well as from their closest known species *M. goodii* (Brown *et al*., 1999). Rather, that tree suggested that strain JS623 was related to *M. moriokaense*. This coincides with the observation made by Coleman and Spain, 2003, that strain JS623 is, based on 16S rRNA gene identity, closely related to another environmental isolate, strain JS619, which in turn was previously classified as being probably *M. moriokaense* (Coleman *et al*., 2002).

The sequence of the 16S rRNA gene is frequently used for species identification in mycobacteria (Tortoli, 2012). However, employing it as the sole criterion for classification is insufficient as the same 16S rRNA gene sequence has been detected in different species (i.e. *M. gastri* and *M. kansasii*; Brown *et al*., 1999) and, on the other hand, more than one sequence has been detected in the same genome of a single species (i.e. *M. celatum*; Menendez *et al*., 2002). Thus, it is considered that the discriminative power of the 16S rRNA gene alone is limited, and more robust phylogenies can be obtained by building a tree with concatenated sequences of several housekeeping genes (Gadagkar *et al*., 2005; Tortoli, 2012). We used this strategy to zoom into the clade comprising the thermo‐tolerant non‐pigmented species plus the thermo‐tolerant pigmented *M. phlei*, as the last also seems to be related to strain JS623 on the basis of the 16S rRNA gene sequences (Fig. 1). To build the tree of concatenated genes, only partial sequences could be compiled of the 16S rDNA, *hsp65*, *rpoB* and *tuf* sequences from *M. smegmatis* candidate strains, including the type strain and selected thermo‐tolerant rapid growers (Supporting Information Table S2). The *M. smegmatis* type strain could be included in this genotype analysis, the only method with sufficient available data for that strain.

**Figure 2:**
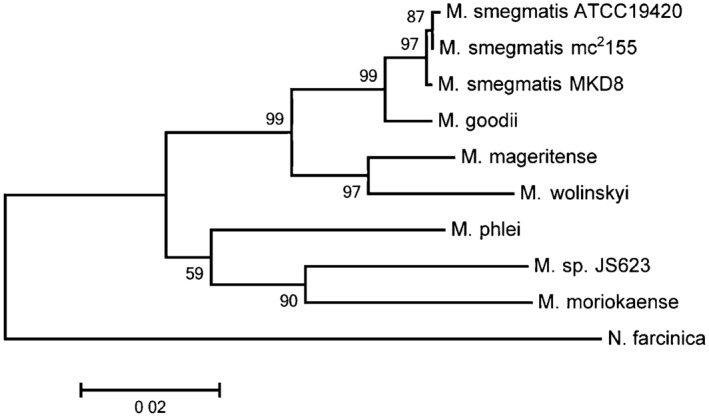
Molecular phylogenetic analysis based on concatenation of partial 16S rRNA, hsp65, rpoB and tuf gene sequences. Numbers are the percentage of trees in which the associated taxa clustered together in the bootstrap analysis. The tree is drawn to scale and the bar length indicates 0.02 substitutions per site. See Supporting Information Table S2 for accession number of the genes and Experimental Procedures in Supporting Information for details.

The concatenated tree substantiates the phylogenetic distance of JS623 to *M. smegmatis* and, again, locates this strain closest to *M. moriokaense* and *M. phlei* (Fig. 2). Contrary to JS623, the other two strains labelled as *M. smegmatis* clustered as expected with the *M. smegmatis* type strain (Fig. 2). The species clustering derived from our concatenated tree agreed with other previously published concatenated trees of the genus *Mycobacterium* (Devulder *et al*., 2005).

### Genome‐scale comparisons

Determining the relatedness of whole genomes should result in a better resolution of the phylogenetic relationship than single and multiple gene identities (Richter and Roselló‐Mora, 2009). This principle has always been taken into account when determining whole genomic DNA relatedness by the traditional wet‐lab DNA–DNA hybridization (DDH), which is still considered the gold‐standard for species delimitation of bacteria, including the genus *Mycobacterium* (Goris *et al*., 2007; Ramasamy *et al*., 2014). Nowadays, WGS offer different and global approaches to support species delineation.

We have compared the sequences of three independent isolates that are listed in the databases as *M. smegmatis* and for which a WGS is available, i.e. strains JS623, mc^2^155 and MKD8. Three different procedures that deliver species identification‐relevant parameters from WGS data were applied: Genome to Genome Distance Calculator (GGDC), Average Nucleotide Identity (ANI), and gene synteny. The genome of *M. phlei* was additionally included in the GGDC and ANI analyses.

GGDC (http://ggdc.dsmz.de) is a web‐based procedure that was established to replace the so far gold standard for species delimitation, DDH, by *in silico* genome‐to‐genome comparison (dDDH). Two genomes are considered to belong to the same species when their dDDH value is higher than 70%, which corresponded to the genome distance value (GD) lower than 0.0258 (Auch *et al*., 2010; Meier‐Kolthoff *et al*., 2013).

The ANI procedure compares the nucleotide sequences of conserved shared genes between two genomes (Konstantinidis and Tiedje, 2005; http://enve‐omics.ce.gatech.edu/ani/). The currently accepted cut‐off value indicating that genomes derive from the same species is about 95% ANI value, corresponding to 70% DDH (Goris *et al*., 2007).

Gene synteny analysis determines the order of genes in the genome. It is believed that changes in the gene order are accumulated during genome evolution and species delineation. Gene synteny analysis was performed with the open‐source software MAUVE (Darling *et al*., 2004) (http://darlinglab.org/mauve/). A value of more than 70% of gene rearrangement is considered to indicate that two genomes belong to different mycobacterial species (Garcia‐Betancur *et al*., 2012).

**Figure 3:**
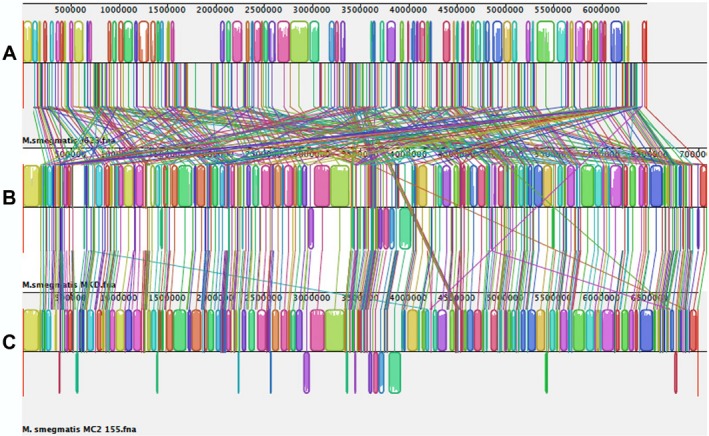
Gene alignment of genomes of the mycobacterial strains using MAUVE multiple alignments. Homologous regions that align to segments of another genome are designated by coloured blocks connected by lines. Genomes of strains from top to bottom: (A) JS623; (B) MDK8; (C) mc^2^155. See Supporting Information Table S2 for accession numbers of the genomes. See text and Experimental Procedures in Supporting Information for details.

The three independent methods used for genome comparison showed a clear separation of JS623 from the other two *M. smegmatis* genomes (Table 2 and Fig. 3). According to the cut‐off values for species delimitation, the strains MKD8 and mc^2^155 belong to the same species, and are at the same time both members of a different species than strain JS623 (Table 2). A differentiation at the species level was also found after comparison with the genome of *M. phlei* (Table 2).

**Table 2:** WGS comparison between *M* *ycobacterium* strain JS623 and other strains

Strain	JS623			mc^2^155		MKD8
	MKD8	mc^2^155	Mph	MKD8	Mph	Mph
% dDDH – GD*	20.3% – 0.21	20.3% – 0.22	21.5% – 0.21	82.5% – 0.021	21.8% – 0.20	21.9% – 0.20
% ANI	81.8%	81.9%	80.1%	98.4%	80.4%	80.8 %
% GR	95%	94%	nd	0.24%	nd	nd

* Percentage of digital DNA–DNA hybridization (dDDH) and correspondent genome distance (GD), percentage of Average Nucleotide Identity (ANI) and percentage of gene rearrangements (GR) between genomes of strain JS623, *M. smegmatis* mc^2^155, *M. smegmatis* MKD8 and *M. phlei* RIVM601174.

Mph, *Mycobacterium phlei*; nd, not done.

See Supporting Information Table S2 for accession numbers of the genomes and text and Experimental Procedures in Supporting Information for further details.

Strains of *M. smegmatis*, namely mc^2^155 and MKD8, showed some differences in their gene order (Table 2 and Fig. 3), concordant with their independent origin (see Supporting Information). Those differences do not include wide gene rearrangements (Table 2 and Fig. 3), which are usually found when comparing genomes from separate mycobacterial species (Garcia‐Betancur *et al*., 2012). On the contrary, extensive segment shuffling was observed when genomes of these two strains were compared with the genome of JS623 (Fig. 3), therefore suggesting that the latter belongs to a different species than *M. smegmatis*.

## Conclusions

The first description of *Mycobacterium* strain JS623 included its preliminary classification as being most similar to *M. smegmatis*, based on the 96.7% identity value of a 421 bp 16S rRNA gene alignment (Coleman and Spain, 2003), and, consequently, the strain was considered in the literature and in several databases (Table 1) as a member of the species *M. smegmatis*. Upon description, the percentage was within the range of species delimitation (95–97%), however, according to the established and currently accepted values afterwards (Ramasamy *et al*., 2014), that percentage is below the species limit of differentiation (98.7%). We reckoned, therefore, that JS623 was probably not a member of *M. smegmatis*. Our subsequently undertaken methodical gene and genome analyses showed that strain JS623 is a mycobacterium more related to *M. moriokaense* than to *M. smegmatis*, and indicate that it is not a member of this last species, as was previously believed. Consequently, and in order to avoid future confusions, the database entries should be corrected accordingly, and JS623 should be referred to as *Mycobacterium* sp. awaiting a formal species description.

The data presented in this work, derived from a multiple approach, underline the strength of genome comparisons to identify erroneously classified genome entries in the databases. Recently, Baez‐Hidalgo *et al*. (2015) detected misclassification of deposited genomes within the genus *Aeromonas* by using a similar combined strategy.

In conclusion, systematic application of the novel *in silico* tools helps to avoid misleading conclusions, as, for example, those derived from comparative genome‐based analyses of mycobacteria, when a genome wrongly attributed to a certain species was included in the analysis.

## Supporting information


**Table S1.** Sources of 16S rRNA gene sequences.
**Table S2.** Sources of gene sequences used in the concatenated tree and genome sequences used in WGS comparisons.
**Appendix S1.** Historical approach to the *M. smegmatis* strains with available WGS data and Experimental Procedures.
