# Supplementary material for: Gene and whole genome analyses reveal that the mycobacterial strain JS623 is not a member of the species M ycobacterium smegmatis
Source: Microb Biotechnol. 2016 Feb 1;9(2):269–74. doi: 10.1111/1751-7915.12336 (PMC4767285; doi:10.1111/1751-7915.12336)
Supplement: Supplementary file 1 — Table S1. Sources of 16S rRNA gene sequences. Table S2. Sources of gene sequences used in the concatenated tree and genome sequences used in WGS comparisons. Appendix S1. Historical approach to the M. smegmatis strains with available WGS data and Experimental Procedures. [file MBT2-9-269-s001.zip › MBT2_12336_Supp0001.pdf]

SUPPORTING INFORMATION

TITLE

Gene and whole genome analyses reveal that the mycobacterial strain JS623 is not a member of the species *Mycobacterium smegmatis*

AUTHOR INFORMATION

Maria J. Garcia[*,1] and Susanne Gola[*,2]

[1] Universidad Autonoma de Madrid, Madrid, Spain.

[2] Centro Nacional de Biotecnología (CNB-CSIC), Madrid, Spain.

* Corresponding authors:

Maria J. Garcia
Department of Preventive Medicine and Public Health and Microbiology
School of Medicine
Autonoma University of Madrid
St/ Arzobispo Morcillo, 4
28029-Madrid
Tfn: +34 914972753
e-mail: mariaj.garcia@uam.es

Susanne Gola
Department of Microbial Biotechnology
National Biotechnology Center (CNB-CSIC)
St/ Darwin, 3. 28049 Madrid. SPAIN
Tfn. +34 915854686
Fax. +34 915854506
e-mail: sgola@cnb.csic.es

Historical approach to the *M. smegmatis* strains with available WGS data.

The most frequently used *M. smegmatis* strain is mc^2155, because it is easily transformable with replicating plasmids, a property called *ept* phenotype (*e*fficient *p*lasmid *t*ransformation, Snapper *et al*., 1990). The history of this popular strain appears to go back to the pioneer era of tuberculosis research in the laboratory of Robert Koch in Berlin from which a mycobacterial strain was sent to F.G. Novy and V. C. Vaughan at the University of Michigan, USA. That strain was stocked as ATCC 607 with the description of being the "Original Koch strain of *Bacillus tuberculosis*". The erroneous belief that ATCC 607 was *Mycobacterium tuberculosis* was clarified in a correspondence between Hastings and McCarter (1932) and Dr. Novy (Hastings and McCarter, 1932). The strain ATCC 607 was renamed as mc^21 by W. R. Jacobs and sub-culturing steps led, through mc^26 and mc^2154, finally to the isolation of the *eptC* mutant mc^2155 (Jacobs, 2014; Panas *et al*., 2014). Contrary to the type strain, whose genome is not available so far, the genome of mc^2155 has been sequenced more than once (http://www.ncbi.nlm.nih.gov/genome/genomes/1026).

The strain JS623 was obtained from soil in 2003 during an investigation on ethene- and vinyl chloride-degrading mycobacteria (Coleman and Spain, 2003) and has been used in metabolic and bioremediation studies of these compounds (Jin and Mattes, 2008; Jin *et al*., 2010). By means of aligning a 421 bp fragment of the 16S rDNA, the closest species to JS623 was determined to be *M. smegmatis* (96.7% identity) (Coleman and Spain, 2003). That percentage of identity was close to, but lower than, the accepted cut-off for species differentiation in the genus (Ramasamy *et al.*, 2014). Since then, strain JS623 has been referred to in the literature either as *Mycobacterium* sp. (Jin *et al.*, 2010) or as *M. smegmatis* (Prasanna and Mehra, 2013). The genome of this strain was made available in December 2012 (Table S2) and added as belonging to *M. smegmatis* to several databases (see Table 1main text).

Another *M. smegmatis* strain with a recently fully sequenced genome is the conjugation recipient MKD8 (Gray *et al.*, 2013b; Table S2). The primordial parent was the Japanese *M. smegmatis* strain PM5 (Tokunaga *et al*., 1973; Mizuguchi *et al*., 1976). A lysine auxotrophic mutant of PM5, strain P73, was obtained in 1972 by Y. Mizuguchi and stocked in W. R. Jacobs'

laboratory as $mc^2$874 (personal communication M. Pavelka; Pavelka and Jacobs, 1996). An *ept* derivative of mc^2 874, strain mc^2 1211, is the direct precursor of strain MKD8 (Parsons *et al*., 1998).

Experimental Procedures

Gene-scale comparisons.

Relationships of gene sequences were estimated using the MEGA6 programme (Tamura *et al*., 2013). Multiple sequence alignments were built by the implemented ClustalW (Thompson *et al*., 1994) with default settings for non-coding DNA and trees inferred by the Maximum-Likelihood (ML) method (Felsenstein, 1981) using *Nocardia farcinica* as the outgroup. The most suited substitution model was identified by the MEGA6 “find best model” function. All positions with gaps were eliminated from the analyses and reliability of trees was assessed by 1.000 bootstrap replications (Felsenstein, 1985).

For inference of the phylogenetic relationship of 25 fast growing mycobacteria based on the 16S rRNA coding sequence, the Tamura-Nei distance model (Tamura and Nei, 1993) with Gamma-distributed rates plus Invariant Sites (TN93+G+I) was used.

For the phylogenetic relationship of the thermo-tolerant non-pigmented species plus *M. phlei* a concatenated tree was inferred based on the General Time Reversible model with Gamma-distributed rates (GTR+G). The partial gene sequences comprised nucleotides 48–589 (16S rDNA), 154–574 (*hsp65*), 1.114–1.508 (*rpoB*), and 299–947 (*tuf*), corresponding to the numbering of the *M. smegmatis* mc^{2}155 genes.

Genome-scale comparisons.

Up to three different procedures were used to perform the WGS comparisons. The methods compare the full-length nucleotide sequence by BLAST alignment (GGDC); the percentage of nucleotide identity between conserved genes (ANI) and the changes in the gene synteny (MAUVE alignment).

1) The method GGDC is based on BLAST nucleotide comparison of entire sequences and calculates digital DDH (dDDH), which allows to specify genome distances. GGDC analyses were run following the alignment method GGDC 2.0 BLAST+ with settings of the recommended formula two (http://ggdc.dsmz.de). This web service allows to infer whole-genome distances able to mimic wet lab DDH.

2) To determine the ANI value between genomes, the two-way calculator was applied using the settings recommended by default to estimate the ANI between each couple of genomic datasets (http://enve-omics.ce.gatech.edu/ani/).

3) Genomes were aligned with the open-source MAUVE aligner, version 2.3.1. Briefly, MAUVE performs a procedure for multiple whole-genome alignments to detect rearrangements and inversions among genomes (http://darlinglab.org/mauve/). During the alignment process, the programme identifies regions without genome rearrangements (named Local Colinear Blocks – LCBs) that serve as anchors for the alignment. Quantitative determination of the genomic order was undertaken by using a backbone file generated according to the method described previously (Garcia-Betancur *et al*. 2012).

The cut-off values considered for species separation are: %dDDH <70% (http://ggdc.dsmz.de); ANI <95% (http://enve-omics.ce.gatech.edu/ani/); % Gene rearrangement >70% (Garcia-Betancur *et al*. 2012). Comparisons made between identical genomes gave in all the cases: dDDH=100% that inferred a GD=0.000; ANI=100%; GR=0%.

References included in Supporting Information

Coleman, N.V. and Spain, J.C. (2003) Distribution of the coenzyme M pathway of epoxide metabolism among ethene- and vinyl chloride-degrading *Mycobacterium* strains. *Appl Environ Microbiol* **69**: 6041-6046.

Felsenstein, J. (1981) Evolutionary trees from DNA sequences: A maximum likelihood approach. *J Mol Evol* **17**: 368-376.

Felsenstein, J. (1985) Confidence limits on phylogenies: An approach using the bootstrap. *Evolution* **39**: 783-791.

Garcia-Betancur, J.C., Menendez, M.C., Del Portillo, P. and Garcia, M.J. (2012) Alignment of multiple complete genomes suggests that gene rearrangements may contribute towards the speciation of Mycobacteria. *Infect Genet Evol* **12**: 819-826.

Gray, T.A., Palumbo, M.J. and Derbyshire, K.M. (2013b) Draft genome sequence of MKD8, a conjugal recipient *Mycobacterium smegmatis* strain. *Genome Announc* **1**, e0014813.

Hastings, E.G. and McCarter, J. (1932) Misnamed cultures and studies of the tubercle bacillus. *Science* **75**: 513-515.

Jacobs, W.R., Jr. (2014) Gene transfer in *Mycobacterium tuberculosis*: Shuttle phasmids to enlightenment. In *Molecular genetics of mycobacteria*, pp.3-25. Edited by G. F. Hatfull and W. R. Jacobs, Jr.. Washington, DC: ASM Press.

Jin, Y.O., Cheung, S., Coleman, N.V. and Mattes, T.E. (2010) Association of missense mutations in epoxyalkane coenzyme M transferase with adaptation of *Mycobacterium* sp. strain JS623 to growth on vinyl chloride. *Appl Environ Microbiol* **76**: 3413-3419.

Jin, Y.O. and Mattes, T.E. (2008) Adaptation of aerobic, ethene-assimilating *Mycobacterium* strains to vinyl chloride as a growth substrate. *Environ Sci Technol* **42**: 4784-4789.

Mizuguchi, Y., Suga, K. and Tokunaga, T. (1976) Multiple mating types of *Mycobacterium smegmatis*. *Jpn J Microbiol* **20**: 435-443.

Panas, M.W., Jain, P., Yang, H., Mitra, S., Biswas, D., Wattam, A.R. *et al.* (2014) Noncanonical SMC protein in *Mycobacterium smegmatis* restricts maintenance of *Mycobacterium fortuitum* plasmids. *Proc Natl Acad Sci U S A* **111**: 13264-13271.

Parsons, L.M., Jankowski, C.S. and Derbyshire, K.M. (1998) Conjugal transfer of chromosomal DNA in *Mycobacterium smegmatis*. *Mol Microbiol* **28**: 571-582.

Pavelka, M.S., Jr. and Jacobs, W.R., Jr. (1996) Biosynthesis of diaminopimelate, the precursor of lysine and a component of peptidoglycan, is an essential function of *Mycobacterium smegmatis*. *J Bacteriol* **178**: 6496-6507.

Prasanna, A.N. and Mehra, S. (2013) Comprehensive phylogenetic analysis of mycobacteria. Preprints of the 12th IFAC Symposium on Computer Applications in Biotechnology, 107-112.

Ramasamy, D., Mishra, A.K., Lagier, J.C., Padhmanabhan, R., Rossi, M., Sentausa, E. *et al.* (2014) A polyphasic strategy incorporating genomic data for the taxonomic description of novel bacterial species. *Int J Syst Evol Microbiol* **64**: 384-391.

Snapper, S.B., Melton, R.E., Mustafa, S., Kieser, T. and Jacobs, W.R., Jr. (1990) Isolation and characterization of efficient plasmid transformation mutants of *Mycobacterium smegmatis*. *Mol Microbiol* **4**: 1911-1919.

Tamura, K. and Nei, M. (1993) Estimation of the number of nucleotide substitutions in the control region of mitochondrial DNA in humans and chimpanzees. *Mol Biol Evol* **10**: 512-526.

Tamura, K., Stecher, G., Peterson, D., Filipski, A. and Kumar, S. (2013) Mega6: Molecular evolutionary genetics analysis version 6.0. *Mol Biol Evol* **30**: 2725-2729.

Thompson, J.D., Higgins, D.G. and Gibson, T.J. (1994) Clustal W: Improving the sensitivity of progressive multiple sequence alignment through sequence weighting, position-specific gap penalties and weight matrix choice. *Nucleic Acids Res* **22**: 4673-4680.

Tokunaga, T., Mizuguchi, Y. and Suga, K. (1973) Genetic recombination in mycobacteria. *J Bacteriol* **113**: 1104-1111.

Supplementary Table S1. Sources of 16S rRNA gene sequences.

organism	strain ([1])	GenBank assembly ID	accession number ([2])
M. abcessus	ATCC 19977 [T]	GCA_000069185.1	NC_010397.1
M. aromaticivorans	JS19b1 [T]	GCA_000559085.2	(contigs)
M. austroafricanum	DSM 44191 [T]	GCA_000612725.1	(scaffolds)
M. chelonae	strain 1518	GCA_000523895.1	(contigs)
M. chubuense	NBB4	GCA_000266905.1	NC_018027.1
M. cosmeticum	DSM 44829 [T]	GCA_000613185.1	(contigs)
M. farcinogenes	DSM 43637 [T]	GCA_000723385.1	(scaffolds)
M. fortuitum	DSM 46621 [T]	GCA_000295855.1	(scaffolds)
M. gilvum	PYR-GCK	GCA_000016365.1	NC_009338.1
M. goodii	ATCC 700504 [T]		AY457079.1
M. hassiacum	DSM 44199 [T]	GCA_000379865.1	(scaffolds)
M. iranicum	UM-TJL	GCA_000455165.1	(contigs)
M. mageritense	DSM 44476 [T]	GCA_000612825.1	(contigs)
M. moriokaense	CIP 105393 [T]		NR_115331
M. neoaurum	DSM44074 [T]	GCA_000724065.1	(scaffolds)
M. phlei	RIVM601174	GCA_000257725.1	(contigs)
M. rhodesiae	NBB3	GCA_000230895.3	NC_016604.1
M. septicum	DSM 44393 [T]	GCA_000455325.1	(scaffolds)
M. smegmatis	mc^{2}155	GCA_000015005.1	NC_008596.1([3])
"M. smegmatis"	JS263	GCA_000328565.1	NC_019966.1([3])
M. smegmatis	MKD8	GCA_000331165.1	(chromosome) NZ_CM001762.1([3])
M. thermoresistibile	ATCC 19527 [T]	GCA_000234585.2	(contigs)
M. vaccae	ATCC 25954	GCA_000295825.1	(contigs)
M. vanbaalenii	PYR-1 [T]	GCA_000015305.1	NC_008726.1
M. wolinskyi	ATCC700010 [T]		NR_042922
Norcardia farcinica	IFM10152	GCA_000009805.1	NC_006361.1

([1]) type strains are indicated by [T]

([2]) for yet unfinished genome projects, i.e. without annotations, the stage of the whole genome shotgun sequence in which the 16S rRNA gene was searched manually is given in brackets

([3]) sequences used for genome-scale comparisons

Supplementary Table S2. Sources of gene sequences used in the concatenated tree and genome sequences used in WGS comparisons.

species	strain ([1])	Genes used in the concatenated tree			
		16S rRNA	*hsp*65	*rpo*B	*tuf*
M. goodii	ATCC 700504 [T]	AF547930	AF547839	AY544918	DQ986127.1
M. mageritense	DSM 44476 [T]	AF547944	AF547853	AY544941	DQ986126.1
M. moriokaense	CIP 105393 [T]	AF547948	AF547857	AY544945	DQ986072.1
M. phlei	RIVM601174	AF547957	AF547866	AY544954	DQ986106.1
M. smegmatis	mc[2]155	NC_008596.1			
M. "smegmatis"	JS263	NC_019966.1			
M. smegmatis	MKD8	NZ_CM001762.1			
M. smegmatis	ATCC 19420 [T]	AF547967	AF547876	AY544964	DQ986129.1
M. wolinskyi	ATCC700010 [T]	AF547981	AF547890	AY544978	DQ986118.1
Norcardia farcinica	IFM10152	NC_006361.1			

([1]) type strains are indicated by [T]
